# Study comparing 3 hour and 24 hour post-operative removal of bladder catheter and vaginal pack following vaginal surgery: a randomised controlled trial

**DOI:** 10.1186/s12905-017-0431-x

**Published:** 2017-09-11

**Authors:** Priya Rajan, S. Soundara Raghavan, Deepak Sharma

**Affiliations:** 1 Department of Obstetrics and Gynaecology, JIPMER, Puducherry, India; 2 Department of Neonatology, Consulatant Neonatologist, National Institute of medical and sciences, Jaipur, Rajasthan India

**Keywords:** Urinary catheter, Vaginal pack, Removal after 3 h, Removal after 24 h; vaginal surgery

## Abstract

**Background:**

Traditional practice after vaginal hysterectomy was to keep the vaginal pack and urinary catheter for 24 hours post operatively. But there were studies that prolonged cathterisation was associated with urinary infection. So this study was conducted to compare the post operative outcome when the urinary catheter and vaginal pack were removed after 3 hours and after 24 hours after surgery.

**Methods:**

The study was done in the Department of Obstetrics and Gynecology, in a tertiary teaching institute of South India from September 2008 to March 2010. It was a randomised controlled trial involving 200 women undergoing vaginal surgery, who were randomly assigned to 2 groups – catheter and vaginal pack were removed either in 3 h in study group or were removed in 24 h in control group. The outcome of the study were vaginal bleeding, urinary retention, febrile morbidity, and urinary infection.

**Results:**

There was no significant difference between the study and control groups with respect to vaginal bleeding (0 and 1%, *p* = 1), urinary retention (9 and 4%, *p* = 0.15), febrile morbidity (7 and 4%, *p* = 0.35), and urinary infection (26% in each group, *p* = 1.0).

**Conclusion:**

Keeping the urinary catheter and vaginal pack for 24 h following vaginal surgery does not offer any additional benefit against removing them after 3 h.

## Background

Hysterectomy is one of the most commonly performed surgery in women next only to caesarean section. Traditionally hysterectomies were performed by the abdominal route. Although it continues to be the most common approach worldwide, recently vaginal hysterectomies and laparoscopic assisted vaginal hysterectomies are gaining popularity and are now replacing abdominal hysterectomies [1].

Vaginal hysterectomy, like any other surgery, has its own complications, like hemorrhage, intra-operative injury to bladder, ureter and bowel, post-operative febrile morbidity, urinary retention and urinary tract infections [2, 3]. It has been a traditional practice to place a vaginal pack following vaginal surgery to prevent reactionary hemorrhage, which hinders normal urinary voiding. Hence to facilitate urinary drainage post-operatively, bladder is catheterized, but this is accompanied by an increased risk of urinary infection, inconvenience for the patients, higher costs and prolonged hospital stay. Therefore, judicious use of catheterization is important. The duration of catheter and vaginal pack has been reduced over the years but still there are no consensus on minimizing the complications [4].

This study was performed with the intention to compare the complications and outcome when the vaginal pack and bladder catheter are removed 3 h postoperatively as against the standard practice of removing them after 24 h in an attempt to reduce complications and hospital stay.

## Methods

The study was done in the Department of Obstetrics and Gynecology, in a tertiary teaching institute of South India from September 2008 to March 2010.This was a randomized controlled trial involving 2 groups of patients who underwent vaginal surgeries in the Department of Obstetrics and Gynecology. The study was approved by institutional research board (IRB) of Jawaharlal Institute of Post-graduate Medical Education & Research (JIPMER), Puducherry, India (EC Ref # 5_ 2008). All the operating surgeons were explained in detail about the study protocols and the steps of different types of surgeries to be followed. Informed written consent was taken from the patient before enrollment in the study. The study group comprised of 100 patients in whom the bladder catheter and vaginal pack were removed in 3 h and the control group comprised of 100 women in whom they were removed in 24 h.

### Inclusion criteria

All women undergoing vaginal surgery namely Ward Mayo operation; Manchester repair; vaginal hysterectomy and amputation of cervix.

### Exclusion criteria

All women having pre-operative positive urine cultures; elevated renal parameters (Blood urea >40 mg/dl, serum creatinine >1 mg/dl); Comorbid illness-diabetes; Intra operative visceral injury; Kelly’s stitch and consent not given by patient.

On admission to the gynecology ward in the institute, detailed history was taken and clinical examination was done for all women planned to undergo vaginal surgery. Age and menstrual status were noted. They were randomised into two groups based on a computer-generated randomization table.

Group 1 – Removal of bladder catheter and vaginal pack in 3 h.

Group 2 – Removal of bladder catheter and vaginal pack in 24 h.

Blood urea, random blood sugar, urine routine and microscopic examination were done for the patients pre-operatively. All the surgeries were performed following standard steps and procedures. At the end of the surgery, according to the standard practice, patients were catheterized with a Foley catheter and vagina was packed with a ribbon gauze.

According to study protocol, vaginal pack and catheter were removed in 3 h or 24 h in Group 1 and Group 2 respectively. As per the type of intervention, blinding was not possible.

Post operatively the patients were observed for vaginal bleeding defined as significant enough to necessitate repacking or exploration; Urinary retention defined as retention of urine that was not relieved by routine measures to facilitate voiding, and which required recatheterization; febrile morbidity defined as temperature > 38 °C on 2 occasions at least 4 h apart and / or more than 24 h after surgery and urinary infections defined as when microscopic examination of the urine revealed pus cells or when urine culture showed growth of pathogenic organisms [5].

Other parameters observed were:

1. Surgery performed
2. Type of anesthesia used
3. Prophylactic antibiotics
4. Duration of surgery
5. Surgeon- consultant / senior resident / junior resident
6. Organism causing urinary infections in and post-operative patients.

### Sample size calculation

The sample size was calculated based on the expected cases during the one-and-a-half-year study period.

### Statistical analysis

All the data was entered in Microsoft excel sheet and SPSS version 16 for windows was used for analysis. Chi square test was used to analyse the data. When the expected cell count was less than 5, Fisher’s exact test was used. For continuous variable Unpaired Student t test was used and mean ± SD was calculated. *p* < 0.05 was considered statistically significant.

## Results

Two hundred women undergoing vaginal surgery during the period from September 2008 to March 2010 were randomly assigned to 2 groups – catheter and vaginal pack were removed after 3 h in the study group and after 24 h in the control group.

The baseline characteristics of both the groups were comparable except more number of post-menopausal women in study group, more consultant operated cases in control group, and operation time more than 90 min in control group (Table 1). All the women in either of the groups were multiparous. The most common indication for surgery was utero vaginal prolapse. Out of these there were 3 women with procidentia in the study group and 10 in the control group. Vaginal elongation of cervix was the next common indication (7% in study and 5% in control group). There were more women with fibroid uterus in the control group (9%) compared to the study group (1%). Two patients in the study group and one in the control group had fibroid uterus with utero vaginal prolapse.

**Table 1:** Table showing baseline characteristic of the study population

Parameter	Study group (3 h) (*n* = 100)	Control group (24 h) (n = 100)	*P* value
Age > 50 years	44	34	0.15
Mean age ± SD	50 ± 1.8	48 ± 2.4	0.65
Post-menopausal	70	56	0.02
Uterovaginal prolapse	90	84	0.22
Spinal anaesthesia	98	95	0.28
Consultant operated cases	21	36	0.02
Operative time > 90 min	38	53	0.03
Ward Mayo s operation	82	81	0.85
Manchester repair	7	3	0.72
Vaginal hysterectomy	10	13	0.68
Amputation of cervix	1	3	0.85

Among the total subjects studied, 163 (81.5%) underwent Ward Mayo’s operation and 10 (5%) underwent Manchester repair and pelvic floor repair was performed in 173 (86.5%) patients. Vaginal hysterectomy and cervical amputation were more commonly performed in the control group (13 and 3%) than in the study group (10 and 1%) respectively, although the difference was not statistically significant.

Majority of the subjects were operated under spinal anaesthesia (96%). Four patients in the control group had general anaesthesia while none in the study group was administered general anaesthesia. One in each group had epidural anaesthesia.

The experience of the operating surgeon may have an influence on bleeding, retention or urinary infection. It was observed that among the total patients studied, 37.5% were operated by senior residents, 34% were operated by junior residents and 28.5% were operated by consultants. Among the study and control groups, 21 and 36% of women were operated by consultants (*p* = 0.02). There was no effect on the study outcome because of the experience of the operating surgeon.

In 45.5% of the whole group operative time exceeded 90 min. There were more women in the control group (53%) than the study group (38%) who had prolonged surgery (*p* = 0.03). 19% of women in the control group and 24% in the study group were operated within 60 min or less. Hypertension was the commonest medical condition found in the women (9%).

There was no significant difference between the two group when the study outcome parameters were compared (Table 2). Only one patient from the control group had vaginal bleeding following removal of vaginal pack. There was no subject in the study group that developed reactionary vaginal bleeding. But statistically there was no significant difference between both the groups. In the woman who had vaginal bleeding, a bleeding vessel was identified and ligated.

**Table 2:** Table comparing study outcome between the study and control group

Parameter	Study group (3 h) (n = 100)	Control group (24 h) (n = 100)	P value
Vaginal Bleeding	0	1	1
Urinary Retention	9	4	0.15
Urinary re – catheterized	10	4	0.09
Febrile morbidity	7	4	0.35
Urinary Infection	26	26	1

Thirteen subjects (6.5%) developed urinary retention post operatively following catheter removal. In the study group, 9% developed retention, whereas in the control group, 4% developed urinary retention, however the difference was not statistically significant (*p* = 0.15).

In our institute, when a patient develops urinary retention, she is examined to ascertain if there is fever, if a perineorrhaphy has been done, if there is local edema and inflammation and if any evidence of urinary infection is present. She is given an analgesic for pain relief, is encouraged to ambulate and is tried to void in the presence of running water and use of local heat for lower abdomen or over perineorrhaphy site. Inspite of these measures if she is unable to void, and if bladder is distended, re-catheterization is done. Fourteen (7%) subjects needed recatheterization among the total subjects. Ten percent in the study group and 4% in the control group were recatheterized subsequent to urinary retention, the difference not being statistically significant (*p* = 0.09). One patient in the study group was recatheterized for monitoring output though she did not have urinary retention because her output was very less post-operatively.

Among all the subjects studied, 11(5.5%) had fever. Seven percent in the study group had febrile morbidity in comparison with 4 % in the control group. However, the difference was not statistically significant (*p* = 0.35)**.**

All women had post-operative urine culture done. When significant growth of organism was identified, urinary infection was diagnosed. The occurrence of urinary infection was high (26%). Both the study and the control groups had urinary infection in 26 women each. E.coli was the commonest organism isolated from urine (54%). Klebsiella and Acinetobacter were found in 11% and 8% respectively. Six percent of the women had polymicrobial infection, while the remaining patients showed other organisms.

No vault infection was detected in either the study or control groups during the stay in the hospital. None of the women reported with vault infection subsequently.

Additional benefits of early removal of the catheter and vaginal pack included early mobilisation and better confidence though these were not measured and analysed.

## Discussion

This study was performed to compare the outcome when the vaginal pack and the catheter were removed 3 h and 24 h after vaginal surgery. Traditional practice of keeping the catheter and pack for prolonged duration post operatively has changed with time, as it became evident that prolonging the duration did not have any additional benefit. Previously studies have been performed comparing catheter removal after 1 day and 3 days. There have also been studies in which one group had their urinary catheters removed immediately after surgery. But this had the disadvantage of difficulty in immediate ambulation and voiding due to the residual effect of regional anaesthesia. The effect of spinal opioids on the bladder lasts on an average for 250 to 300 min, which includes the surgery duration and post-operative time for recovery of bladder function. Considering this we have chosen to remove the catheter at 3 h post operatively allowing time for recovery [6]. To overcome this difficulty, the catheter was removed 3 h after surgery in this study. Vaginal packing has not been studied by many workers.

Glavind et al. reported vaginal bleeding in 2.9% of women in whom vaginal pack was removed after 24 h and no bleeding among the women in whom the pack was removed after 3 h [5]. In our study, one patient in the control group (24 h group) had bleeding and early removal of the pack did not result in more incidence of vaginal bleeding.

Most of the studies compared removal of the catheter 24 h post operatively with no catheterization [7–10]. Hakvoort et al. compared catheter removal in 24 h with removal after 5 days [11]. In our study, outcome was compared between 3 h and 24 h removal of the catheter.

Retention rate was more in the prolonged catheterization group in studies by Summitt et al. [7] and Sekhavat et al. [9] In studies by Glavind et al. [5], Dunn et al. [8], Liang et al. [10] and Hakvoort et al. [11], the retention rates were more in the early removal group as compared to the delayed removal group which is comparable to our present study.

Liang et al. reported very high need for recatheterization in their series – 38% when no catheter was introduced, 12% when catheter was kept for 24 h and 10% when catheter was kept for 48 h [10]. All the patients in their study underwent laparoscopic assisted vaginal hysterectomy under general anaesthesia and it is difficult to explain such frequent need for recatheterization. Hakvoort et al. reported 40% need for recatheterization in women who underwent anterior colporrhaphy if the catheter was removed in 24 h [11].

The study conducted by Summitt et al^.^ found higher incidence of post-operative febrile morbidity when catheter was kept longer (24.5% vs 8%) [7]. In the study by Dunn et al. [8] and in the present study, there was no increase in the febrile morbidity when the catheter was kept for longer duration (4% vs 4.8% and 7% vs 4% respectively).

Most of the studies reported higher incidence of urinary infection when the urinary catheter was kept for longer time [5, 9–11]. Paradoxically Summitt et al. reported higher incidence of infection in women in whom post-operative indwelling catheter was not left [7]. Though our study found high incidence of urinary infection in both the groups, there was no difference between the study and control groups. This is similar to the findings of Dunn et al. [8].

The strengths of this study are that it is a prospective randomised study with a large sample size and novelty in terms of investigating the removal of bladder catheter and vaginal pack following vaginal surgery. In this study, we tried to reduce vaginal pack and urinary catheter time. The study had well-defined inclusion and exclusion criteria with well-defined study protocols. All the surgeon in the department followed the study protocol thus reducing the chances of bias. The limitations of the study are that it included several types of surgery (though the numbers were small), different surgeons operating, and use of general anaesthesia in some cases.

## Conclusion

Keeping the vaginal pack and indwelling catheter for a shorter period of 3 h did not result in increase in vaginal bleeding, retention of urine, febrile morbidity and urinary infection. Even though in our study prolonged catheterization was not associated with increased fever or urinary infection, in view of other studies reporting significant increase in urinary infection with prolonged catheterization, it may be prudent to remove the vaginal pack and catheter after 3 h giving allowance to the possible effect of anaesthesia on urinary function.

## Abbreviations

IRB: Institutional research board
